# Monitoring paddy productivity in North Korea employing geostationary satellite images integrated with GRAMI-rice model

**DOI:** 10.1038/s41598-018-34550-0

**Published:** 2018-10-31

**Authors:** Jong-min Yeom, Seungtaek Jeong, Gwanyong Jeong, Chi Tim Ng, Ravinesh C. Deo, Jonghan Ko

**Affiliations:** 1 Satellite Operation & Application Center, Korea Aerospace Research Institute, 169-84 Gwahak-ro, Yuseong-gu, Daejeon, 305-806 Republic of Korea; 2 Applied Plant Science, Chonnam National University, 77 Yongbong-ro, Buk-gu, Gwangju, 61186757 Republic of Korea; 3 Department of Geography, Chonnam National University, 77 Yongbong-ro, Buk-gu, Gwangju, 61186 Republic of Korea; 4 Statistics, Chonnam National University, 77 Yongbong-ro, Buk-gu, Gwangju, 61186 Republic of Korea; 5 School of Agricultural, Computational and Environmental Sciences, Centre for Sustainable Agricultural Systems and Centre for Applied Climate Sciences, Institute for Life Science and the Environment, University of Southern Queensland, Springfield, QLD4300 Australia

## Abstract

To meet the growing demands of staple crops with a strategy to develop amicable strategic measures that support efficient North Korean relief policies, it is a desirable task to accurately simulate the yield of paddy (*Oryza sativa*), an important Asian food commodity. We aim to address this with a grid-based crop simulation model integrated with satellite imagery that enables us to monitor the crop productivity of North Korea. Vegetation Indices (VIs), solar insolation, and air temperature data are thus obtained from the Communication Ocean and Meteorological Satellite (COMS), including the reanalysis data of the Korea Local Analysis and Prediction System (KLAPS). Paddy productivities for North Korea are projected based on the bidirectional reflectance distribution function-adjusted VIs and the solar insolation using the grid GRAMI-rice model. The model is calibrated on a 500-m grid paddy field in Cheorwon, and the model simulation performance accuracy is verified for Cheorwon and Paju, located at the borders of North Korea using four years of data from 2011 to 2014. Our results show that the paddy yields are reproduced reasonably accurately within a statistically significant range of accuracy, in comparison with observation data in Cheorwon (*p* = 0.183), Paju (*p* = 0.075), and NK (*p* = 0.101) according to a statistical *t*-test procedure. We advocate that incorporating a crop model with satellite images for crop yield simulations can be utilised as a reliable estimation technique for the monitoring of crop productivity, particularly in unapproachable, data-sparse regions not only in North Korea, but globally, where estimations of paddy productivity can assist in planning of agricultural activities that support regionally amicable food security strategies.

## Introduction

According to the United Nations Food and Agriculture Organization (FAO) and also World Food Program (WFP), North Korea relies heavily on food imports, bi-lateral sources of food assistance, and multi-lateral international food aid, primarily from China and the Russian Federation^1^. The Democratic People’s Republic of Korea is in East Asia and, it borders China, Russia, and South Korea. North Korea lies at high latitudes between 37°N and 43°N and at longitudes between 124°E and 131°E. It has an extended winter period and an only few frost-free days, with single cropping system that dominates the cultivation. Approximately 1.96 M ha (19.5%) of the total area of the country (12.0 M ha) is arable, with 0.32 M ha of perennial crops such as mulberry and fruit^2,3^. Out of the total gross domestic product (GDP) in North Korea, agriculture is the third most important economic factor supporting the nation, which comprises approximately 25.2% of the GDP^3^. Out of the main food crop products, rice is considered to be the most important staple food crop in the country, followed by corn, soybean, and potato^1^.

The shortage of food in North Korea, which eventually evolved into famine in the mid-1990s and even persists today, was the outcome of multiple interacting factors, including agricultural policies influenced by the political and economic situation, as well as weather conditions^4,5^. Abnormal weather conditions and global warming has mainly impacted the farming environment. Today, climate change scenarios indicate that agriculture will predominantly suffer from a deficiency in water resources^6,7^. According to the Korea climate change assessment report^8^, the degree of climate change in East Asia is more severe than the global trends, and North Korea is one of the most vulnerable countries in this region. The agricultural environment in North Korea is therefore likely to further deteriorate with rapidly changing weather conditions, as predicaments of long-term climate change.

An important reason for the agricultural collapse in North Korea has been a lack of sufficient strategic advice and unsustainable national agrarian policies^5^. A lack of farming policy planning is also likely to have contributed negatively to the future of the agricultural industry, leading to a deterioration of gross agricultural productions. A careful monitoring and accurate prediction of the agrarian yield is therefore paramount for the grain aid organisations, helping determine appropriate solutions to the food shortage issue^2^. However, due to a crop field’s inaccessibility due to its remoteness, it is challenging to acquire real-time agricultural information on primary crop productivity, *i*.*e*., the quantitative measure of crop yield in a given measured area of fields, which is the remnant of past and current political issues in North Korea. Also, accurate and real-time assessment of crop damage caused by natural disasters (*e*.*g*., crop defoliation, drought, and pest infestation) is further likely to benefit from well-designed strategic planning measures implemented to meet the crop production demands in this nation^9^.

An integration of crop modelling and remote sensing techniques is a considered as useful scientific ploy for evaluating the crop growth and gross productivity^10^. Crop models are employed to describe the crop conditions during a growing season, but conventional crop models with a relatively significant number of model input requirement, can be inadequate for simulating the specific spatial variations in seasonal crop growth patterns^11^. An advantage of crop models, however, is that the crop simulations can be taken as a pivotal tool for continuous description of crop growth and their developments by simulating the biophysical processes in the soil-crop-atmospheric system, offering significant positive foresights to support agricultural precision^12^. A disadvantage of crop models, though, is that it is impossible to project a detailed topographical information about the fields and incorporate into the model the regional growth variations and initial conditions, and the largely non-universal model parameters, required to simulate crop models in diverse regions. Also, the use of crop modelling alone can be an inadequate decision-support tool for generating the general crop characteristics^13^ and for estimating the regional crop yield when no spatial biophysical and meteorological parameters are available.

To address the aforementioned issues, optical sensor-based remote sensing techniques can provide real-time crop growth information in great detail, and it can also be useful for observing the crop growth to support on-site ground truth studies^14^. Besides, a significant advantage of remote sensing is that, the spatial data can be acquired for any region on the Earth’s surface using a satellite source where the satellite footprint is available. However, a major limitation of the optical sensor-based imaging is the unfavourable atmospheric conditions that restrict the timely crop data retrieval; something that is predominantly caused by cloud cover linked to periodic rainy monsoon seasons in many parts of Asia^15,16^. Crop models combined with remote sensing imageries from operational satellites have therefore been used to assess the crop growth conditions and crop yields at regional scales^9,17–19^. A few different calibration procedures have also been applied to minimize the numerical difference between the model simulations and observations from remote sensing approaches, particularly useful for data sparse, and geographically isolated regions.

Here, we adopt the gramineous crop model, referred hereafter as, GRAMI^10^ that aims to employs remote sensing dataset for crop growth monitoring and yield mapping^11^. The GRAMI model was initially developed to simulate the growth and development of gramineous crops (*i*.*e*., maize, sorghum, and wheat) based on the integration of remote sensing data. A pertinent point of caution is that, the GRAMI simulations of the crop conditions and the respective yields using satellite dataset should carefully consider the spatiotemporal observation points of biophysical parameters, to acquire reliable simulation results for the region of interest. In this regard, temporal point observations alone are not adequate for reflecting the actual crop phenology using indirect satellite indicators such as Normalized Difference Vegetation Index (NDVI). Among the biophysical parameters of a crop model, plant canopy is the most critical factor reflecting the actual crop growth and subsequent development^20^. A continuous profile of the reflectance data of the plant canopy is used to indirectly observe the current growth status of plants through a vegetation index^21^. Plant canopy is more sensitive to the crop simulation results compared to the other variables such as plant biomasses, photosynthetic assimilation, and evapotranspiration. In addition, the time series data of vegetation indices have registered good performance in national scale retrieval of paddy rice agriculture, since a phenology-based approach could reflect the unique profiles of paddy rice VIs which were spatially changeable by climatic and environmental characteristics of each region^22,23^.

In the present context, GRAMI employs a ‘within-season’ calibration procedure that forces the model simulation to fit the observed values using a set of iterative mathematical processes. This procedure manipulates the initial conditions and model parameters that affect the crop growth simulations to minimise the error between simulations and observations from a remotely sensed data source. The ‘within-season’ calibration procedure ensures that infrequent observations can be used to calibrate the model. GRAMI interpolation methods can fill or smooth the noise and sparse remote sensing observations. In a similar manner, it is possible to use temporal smoothing techniques (e.g., Fourier harmonics, threshold methods, and curve-fitting methods)^24–27^. However, it is difficult to simulate crop conditions using sporadic and long-term missing values due to the prevalence of cloudy monsoons during a crop growing season.

North Korea is dominated by a rainy, summer monsoon season^28^. Therefore, it can be problematic to acquire crop canopy dataset in summer growing period using polar orbit satellites, e.g., Moderate Resolution Imaging Spectroradiometer (MODIS) despite the availability of Terra and Aqua sensors with improved temporal resolution. To resolve this issue in the GRAMI crop model, Yeom *et al*.^18^ demonstrated the potential to reproduce rice yield with remotely sensed data from a high sequential resolution satellite. They uniquely applied Geostationary Ocean Color Imager (GOCI) and Meteorological Imager (MI)^29^, as opposed to the MODIS products over East Asia. In this study, we apply the GOCI-based NDVI profiles to monitor rice growth conditions and developments that reflect weather conditions of North Korea in the summer rainy season.

In this research, we also acquire spatial meteorological parameters such as solar insolation and air temperature using satellite measurements and reanalysis data that better reflect agricultural conditions to map the crop yield with GRAMI model for inaccessible North Korean region. As opposed to the vegetation index, solar insolation and air temperature are physical variables that directly influence the crop growth and development because they represent, respectively, the energy source for photosynthesis and environmental controls on vegetation growth and morphogenesis. Although some research works has used ground measurements to estimate the spatial distribution of meteorological parameters using spatial interpolation approaches, this becomes largely restrictive when considering areas with sparse ground measurement networks. North Korea is one such country due to its complex terrain and inaccessibility issues; thus, crop growth must ideally be simulated with meteorological parameters that integrate remote sensing and reanalysis data.

**Figure 1:**
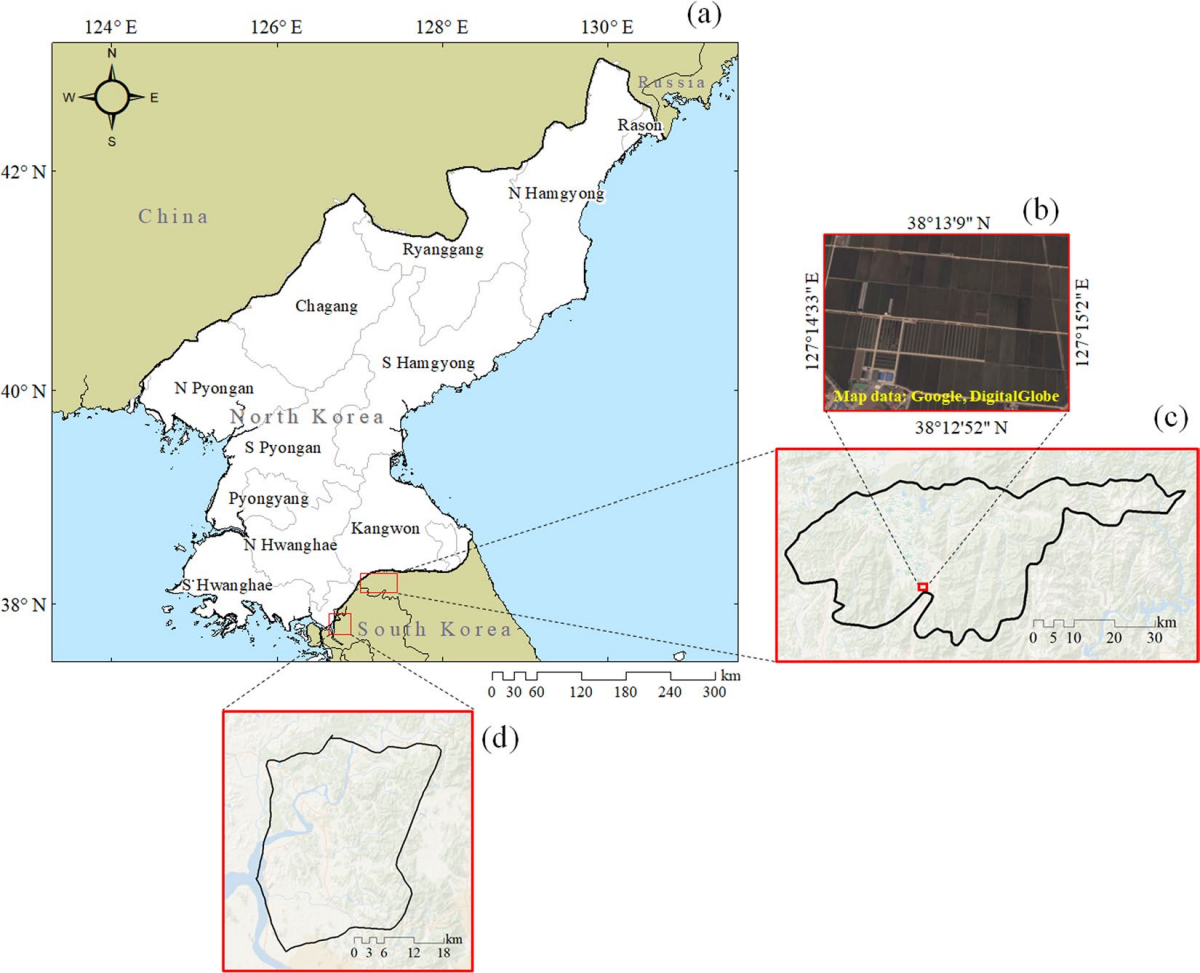
A map of North Korea with the provincial boundaries (**a**) and geographic locations (**b**–**d**) considered in this study in South Korea. The sites include experimental fields in satellite imagery at the branch station of National Institute of Crop Research in Cheorwon (**b**) for parameterization of the initial-condition of the GRAMI model and Cheorwon (**c**) and Paju (**d**) for validation of the model for crop productivity of North Korea. The satellite imagery was obtained from Google Earth Pro 7.3.1 (Google Inc., CA, the USA).

While there have been previous reports about food security issues^4,5^ and also some studies on crop classification and acreage assessments^2^ as well as drought index and gross primary productivity studies^30^ for North Korea, reliable studies on the productivity of staple crops have not been reported yet to the best of the authors’ knowledge. In the current study, we aim to reproduce rice productivity in North Korea with a grid-based crop model combined with satellite imagery, which allows to monitor the crop productivity of North Korea to address the growing demands of staple crops. The monitoring of the agricultural environment in North Korea is undertaken by integrating the GRAMI model with satellite-based biophysical and meteorological parameters based on the calibration of the model using ground truth data from Cheorwon, South Korea (see Fig. 1). This ground truth site has the most similar agricultural environment for rice cultivation to the target region of all available areas surrounding the country. We focus on rice because of the limited ground truth data required to calibrate the model for the other staple crops. The novelity of this research paper is therefore to develop a new satellite-based data-driven tool used to monitor and determine the agronomic environments in the scientifically, politically and socially challenged region of North Korea by integrating the GRAMI model with biophysical and meteorological parameters from the COMS GOCI and MI images.

## Results

### Biophysical and meteorological parameters of North Korea

Prior to simulating crop conditions and estimated yield by a GRAMI model integrated with satellite-based biophysical and meteorological parameters (i.e., NDVI dynamics and solar insolation) we analysed each data product to interpret the reliability of model inputs as precise inputs play an crucial role in the accuracy of the predicted the yield.

**Figure 2:**
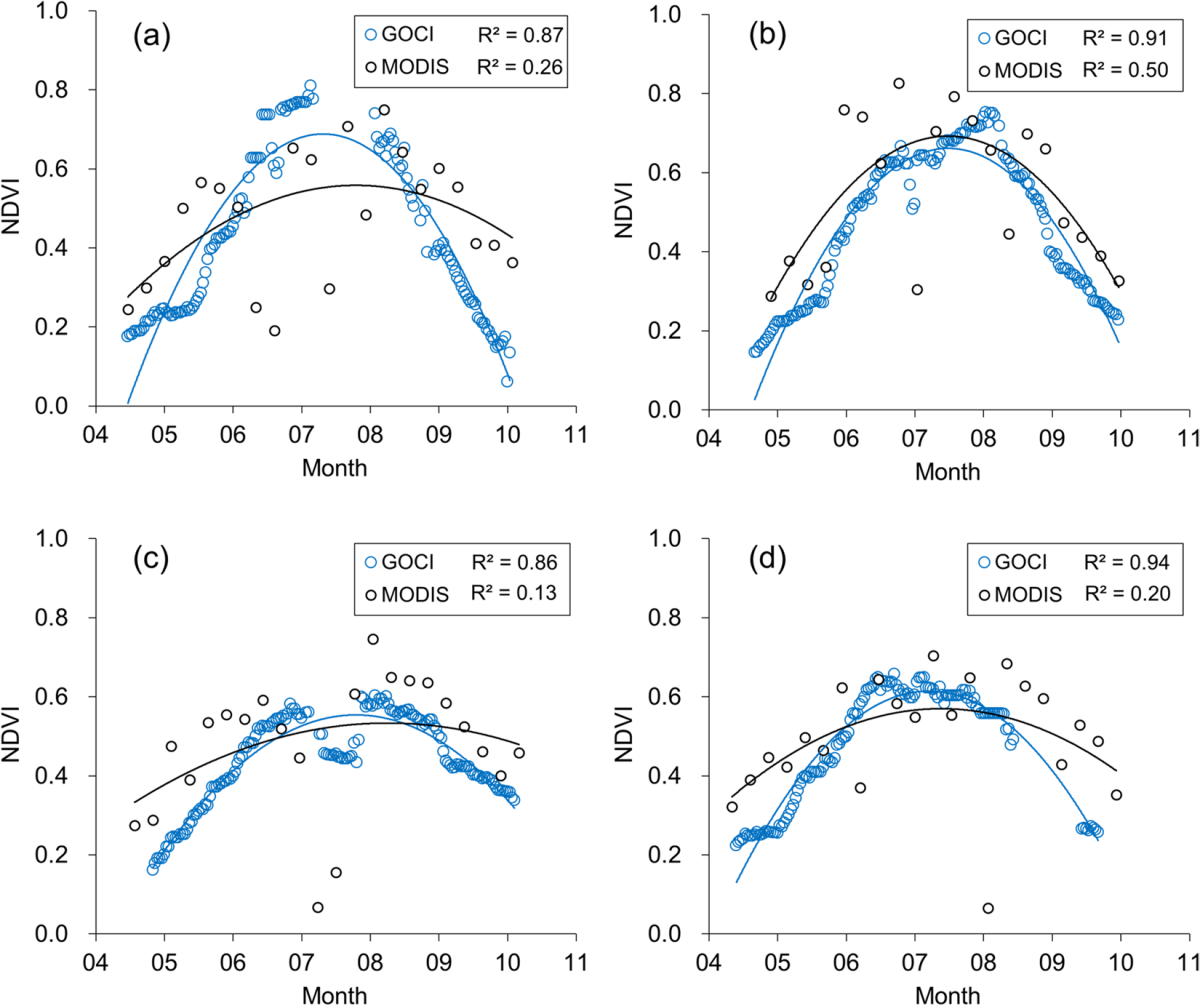
A comparison of the temporal variation of NDVI samples derived from the GOCI and MODIS sensors (i.e., MCD43 8-day product) for the paddy field considered in Paju, South Korea in 2011 (**a**), 2012 (**b**), 2013 (**c**), and 2014 (**d**). The coefficients of determination (R^2^) were computed for the best-fit, quadratic model regression lines of the seasonal variations of the NDVI data.

**Figure 3:**
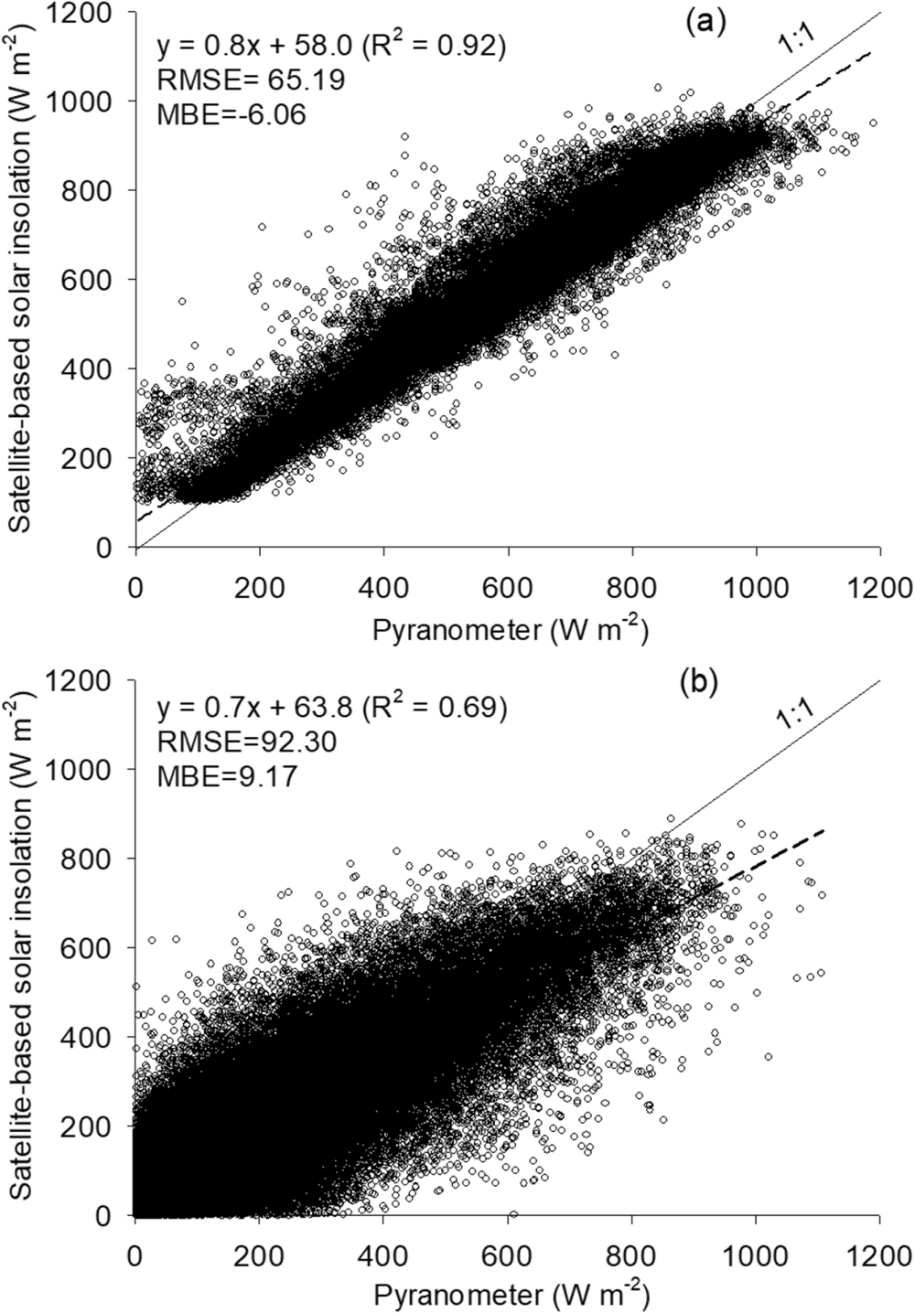
Exploring the relationships between satellite-based solar insolation and land-surface based, pyranometer dataset under clear sky condition (**a**) and cloudy sky condition (**b**) for the study period. Note that the root mean square error (RMSE), mean bias error (MBE) and the linear fit *y* = *mx* + *C* is included in each panel.

In the first stage, we analysed GOCI-based NDVI profiles for selected crop productivity validation sites in the Paju region, South Korea. Figure 2 is a sample plot of annual NDVI profiles from GOCI and MODIS data. GOCI-based NDVI profiles were estimated over a daily temporal resolution. However, in the case of MODIS, we adopted 8-day NDVI profiles from MODIS-based Nadir BRDF Adjusted Reflectance (NBAR) (MCD43) which is officially distributed by NASA. A plausible reason why we used 8-day MODIS NBAR NDVIs from NASA rather than directly estimating daily MODIS products, is to demonstrate the underlying limitations in applying official MODIS NBAR products to the present study area due to a rainy monsoon. Evidently, the GOCI-based BRDF Adjusted Reflectance NDVIs (Blue circles) efficiently describe the annual rice crop development with less scatter, especially during summer monsoon season (June to August) as exemplified in Fig. 2, despite the high rate of cloud cover. Furthermore, MODIS-based NDVI profiles from the NBAR (MCD43) 8-day product is known to exhibit discontinued crop growth and developmental variations, especially for the summer growing period. In respect to the statistical results, for all years, the coefficient of determination (R^2^) for the GOCI BAR NDVI profiles were higher than those of MODIS. One plausible reason for the different NDVI profiles, especially in the rainy monsoon season, is that the GOCI geostationary satellite with more frequent observations, can monitor the phenology of paddy rice more effectively than the MODIS satellite, which can acquire images only twice a day. More details about the BRDF adjusted NDVI profile comparison between GOCI and MODIS over present study area are well documented, for example, in Yeom *et al*.^31^ that also included daily MODIS NDVI profiles comparison with the GOCI values. In the next stage, we validate the COMS MI hourly based insolation with pyranometer data acquired from South Korea, as outlined illustratively in Fig. 3. Owing to the apparent logistic constraints (including a lack of pyranometers) there is no doubt that a validation of satellite-based insolation over North Korea can be challenging. Also, the solar insolation can be more sensitive to atmospheric influences (e.g. cloud cover) relative to regional characteristics, since the rice paddies are largely located on flatlands. Considering this, it was appropriate to utilize pyranometer data based on the adjacent South Korean region to validate the estimated satellite-based insolation.

In Fig. 3, we display the statistical relationship obtained between COMS MI hourly and the observed solar insolation obtained with a pyranometer. Under clear skies (Fig. 3a), a relatively large correlation coefficient (R^2^ ≈ 0.92) is observable, confirmed by a 1:1 line gradient of approximately 0.80. For cloudy conditions, a relatively larger scatter is notable, with generally low values of the solar insolation that are recorded by both, the satellite and the pyranometer attributable to the cloud attenuation effect. The accuracy of the downscaled satellite-based insolation is therefore lower for cloudy conditions, mainly because it is difficult to reflect the complex cloud attenuation effects constrained by the limited COMS MI spectral bands. For comparison purposes, we note that the root mean square error, *RMSE*, (mean bias error) values for clear and cloudy conditions were found to be approximately 65.19 (−6.06) W m^−2^ and 99.30 (9.17) W m^−2^, respectively, indicating a reasonable level accuracy obtained for the GRAMI crop model inputs.

### Paddy field area classification in North Korea

**Figure 4:**
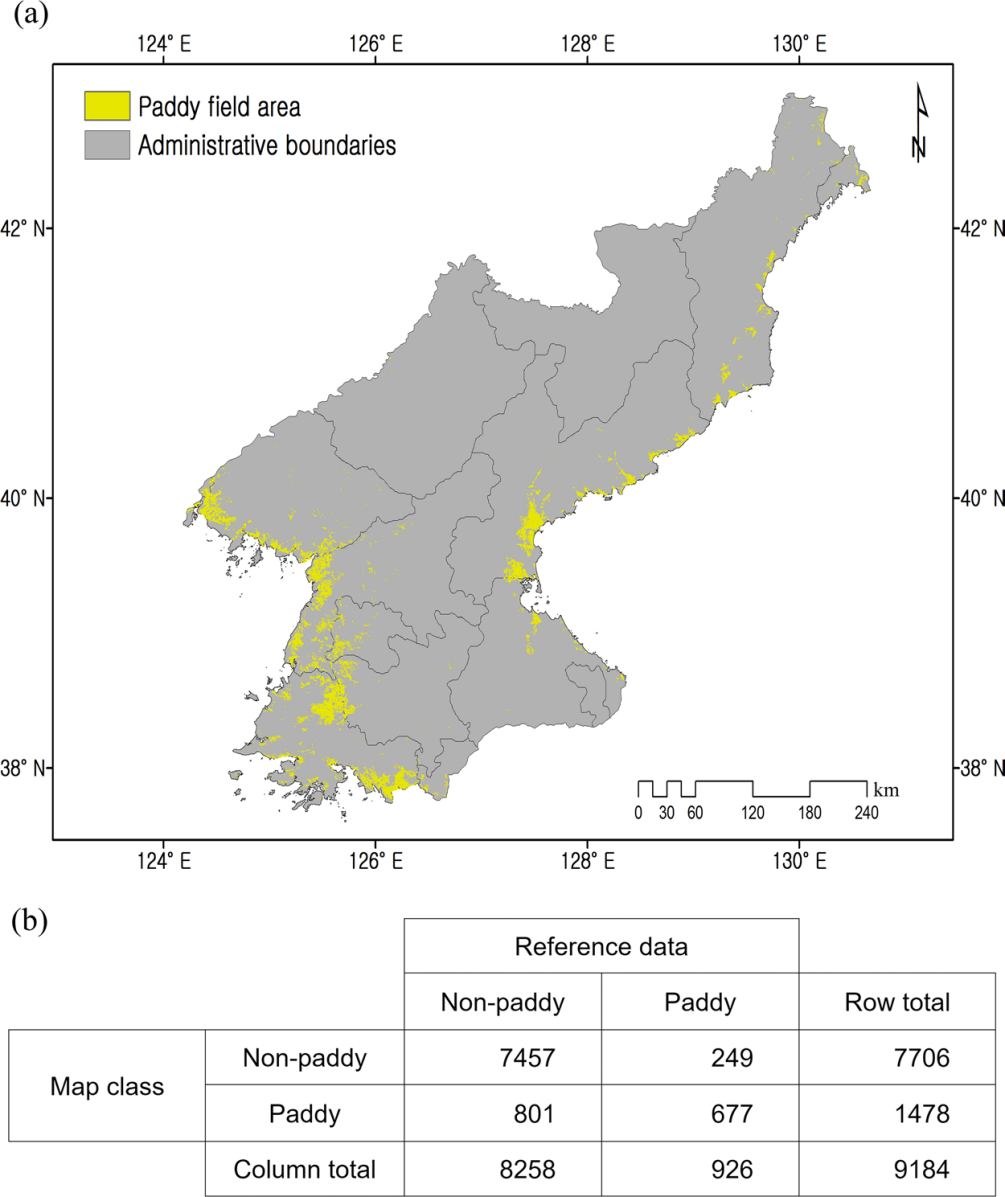
(**a**) The classified paddy field regions located in North Korea. (**b**) The error matrix associated with the paddy area classification map.

The results of the paddy area classification are displayed in Fig. 4. The estimated total area of paddies in North Korea was 491,325 ha and the variables selected by means of the recursive feature elimination (RFE) algorithm were found to be, in order of: wetness index, elevation, and paddy classification index (PCI), maximum peak value, maximum fading date, maximum peak date, maximum fading rate, slope degree, and maximum growth rate. The topographical variables such as wetness index and elevation were therefore chosen as the most important variable used for classifying the paddy areas. RCI also turned out to be an important variable. The overall fractional accuracy of the South Hwanghae validation map was found to be approximately 0.89, with a kappa coefficient of 50.1% (Fig. 4b).

### Paddy productivity in North Korea

**Figure 5:**
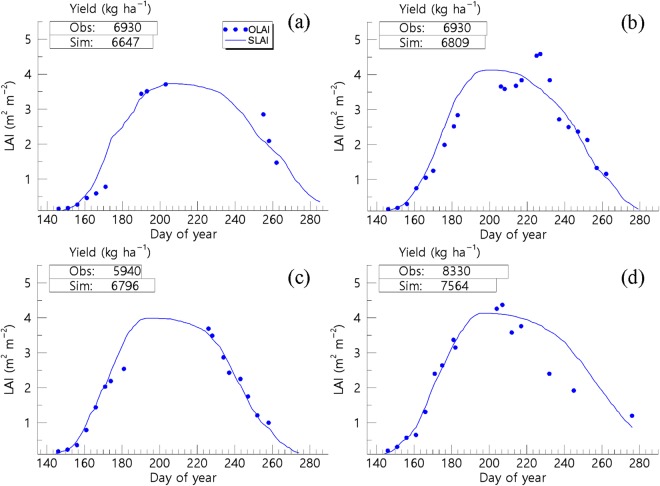
The simulated leaf area index (SLAI) and the respective crop yield in comparison with the observed LAI (OLAI) and the crop yield for the Cheorwon experimental fields in the National Crop Research station, South Korea in (**a**) 2011, (**b**) 2012, (**c**) 2013, and (**d**) 2014. (Note: Obs = observed and Sim = simulated).

In the calibration stage, the simulated values of Leaf Area Index (LAI) and grain yield revealed a statistically acceptable level of agreement with their respective observed values (Fig. 5). In particular, the *RMSE* and model efficiency (*ME*) values were found to be 0.37 m^2^ m^−2^ and 0.93 in 2011, 0.44 m^2^ m^−2^ and 0.90 in 2012, 0.24 m^2^ m^−2^ and 0.95 in 2013, and 0.45 m^2^ m^−2^ and 0.89 in 2014, respectively, for the LAI variable.

According to a two-sample *t*-testing procedure, there was no statistically significant difference (*p* = 0.890) between the simulated and observed paddy yields, since an *RMSE* of 0.59 t ha^−1^ was obtained. In terms of validation, the simulations concurred with observed LAI values where an *RMSE* and *ME* ranges from 2011 to 2014 for Cheorwon was found to be 0.59 to 0.81 m^2^ m^−2^, and −0.06 to 0.29, whereas for Paju, they were 0.42 to 0.67 m^2^ m^−2^ and −0.35 to 0.39. According to a two-sample *t*-testing procedure, the simulated paddy yield did not differ significantly from the observed paddy yield in both Cheorwon (*p* = 0.183) and Paju (*p* = 0.075) and this also concurred with *RMSE* of 1.19 and 0.69 t ha^−1^, respectively.

**Figure 6:**
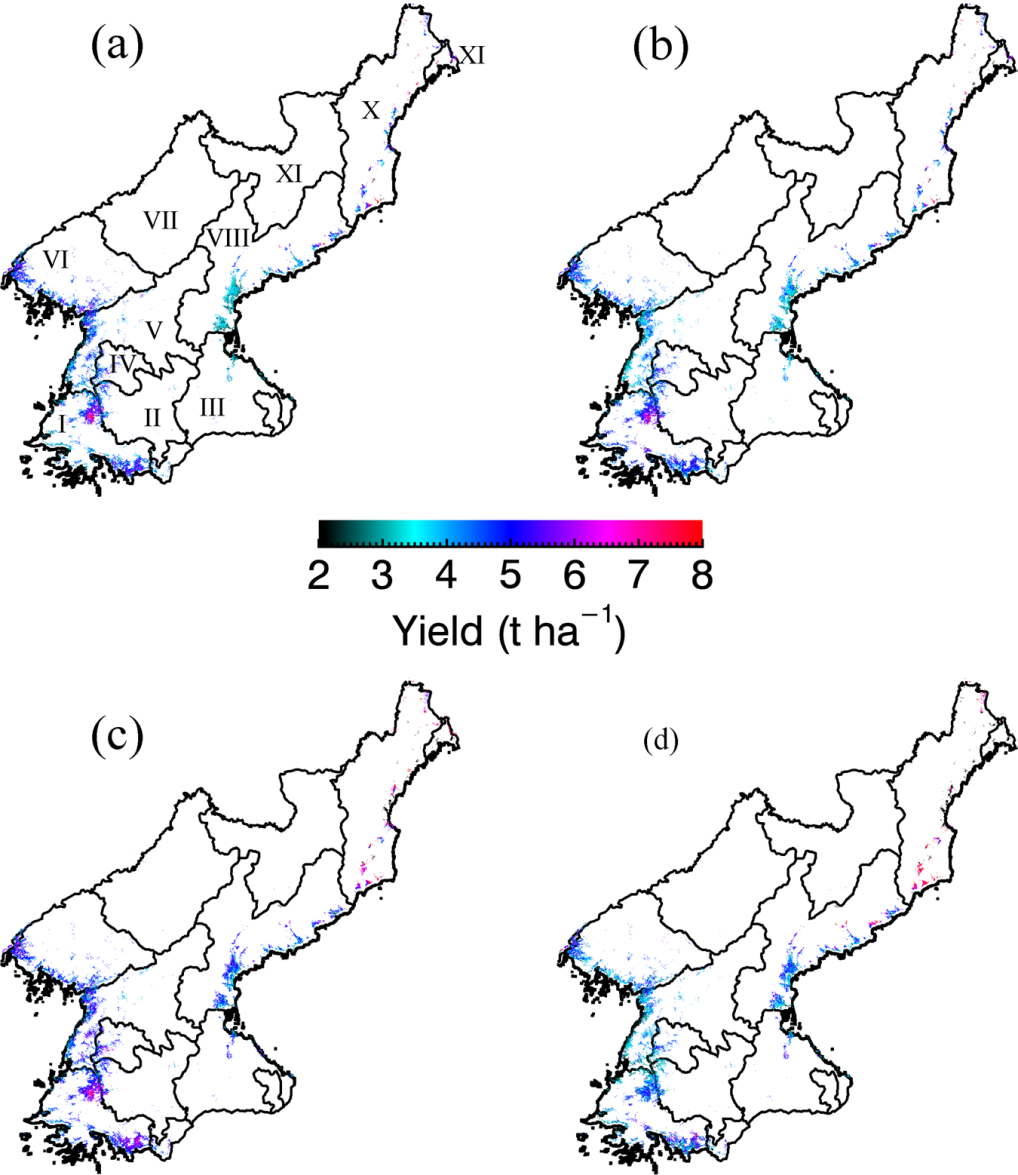
Geographical projections of the simulated values of paddy yields in North Korea in 2011 (**a**), 2012 (**b**), 2013 (**c**), and 2014 (**d**). The yields are projected to only the paddy production regions based on the paddy fields classification (refer to Fig. 4). Provinces are presented as I (S. Hwanghae), II (N. Hwanghae), III (Kangwon), VI (Pyongyang), V (S. Pyongan), VI (N. Pyongan), VII (Chagang), VIII (S. Hamgyong), IX (Ryanggang), X (N. Hamgyong), and XI (Rason).

**Figure 7:**
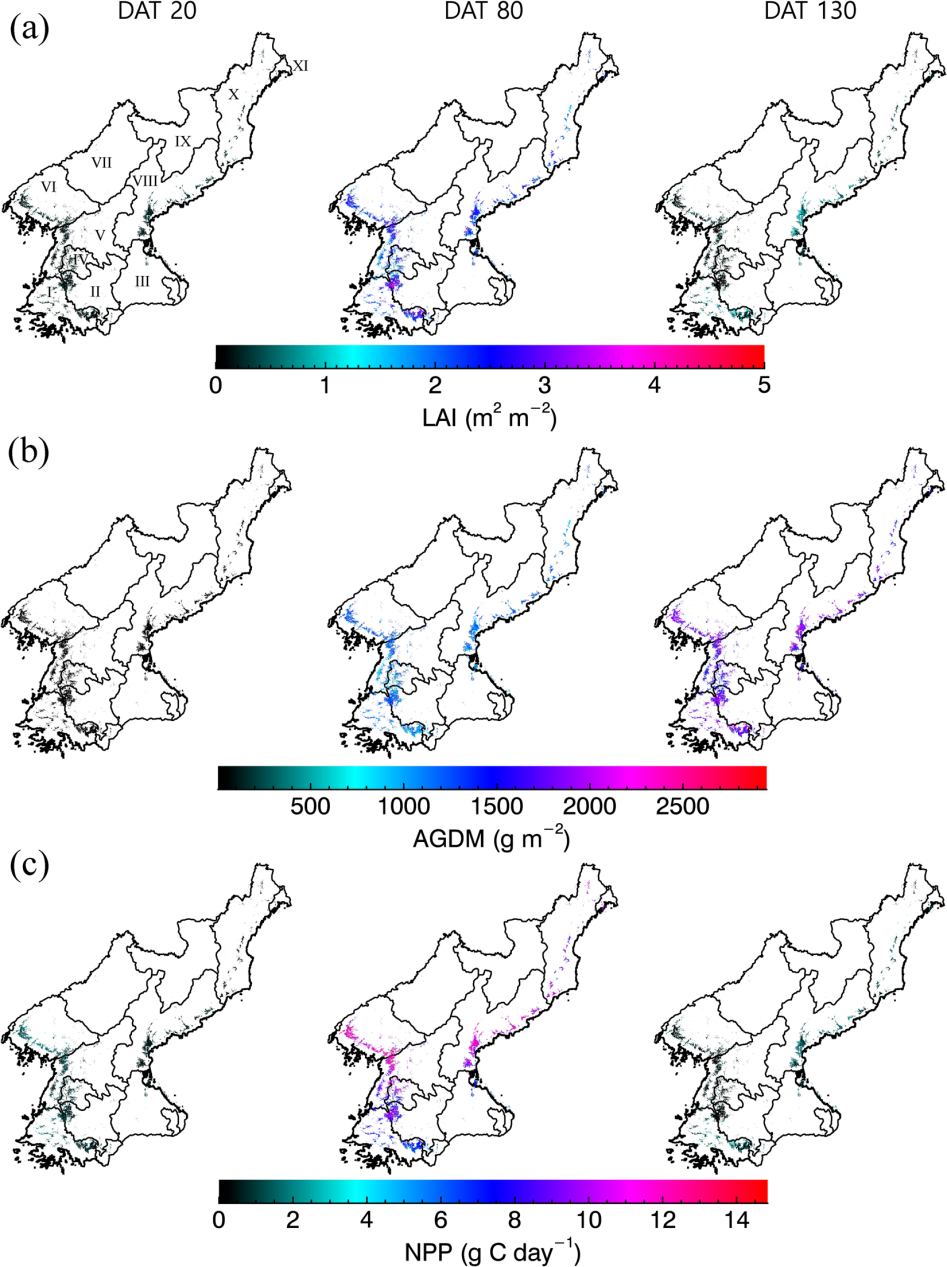
The spatiotemporal projection of simulated values of the leaf area index, LAI (**a**) and the above-ground dry mass, AGDM (**b**), and net primary productivity, NPP (**c**) in North Korea in 2014. The growth variables are projected to only the paddy production regions based on the paddy fields classification (refer to Fig. 4). Pyongyang), V (S. Pyongan), VI (N. Pyongan), VII (Chagang), VIII (S. Hamgyong), IX (Ryanggang), X (N. Hamgyong), and XI (Rason).

Figures 6 and 7 show the projection of the geographical distribution of paddy yield and growth in North Korea the growing season from 2011 to 2014. In Fig. 6, the annual variation in the paddy yield reflects an excellent spatial representation. Highly productive rice paddy areas are mainly located in the Hwanghae province, within the areas classified as the paddy fields in North Korea. According to the FAO report, the annual paddy yield exhibited an increase until the year 2013, before a decline recorded for 2014. By means of Fig. 6, we note that the paddy yield for Hwanghae and Pyongan provinces have decreased in 2014 relative to previous years, indicating that our method is able to simulate the spatiotemporal yield of rice paddies in an efficient manner. This notion is also supported by projected results, where an *RMSE* value of 0.60 t ha^−1^ and a statistically significant result (at *p* = 0.101) was obtained between the simulated and observation data (using the values from FAO report) and a two-sample t-testing procedure.

To further explore the reliabiliy of the projected results we note that the simulated paddy yields in the administrative province of N Hamgyong and S Pyongan registeed significantly discernible values from the observations, at *p* < 0.05 (data not shown) according to two sample t-testing procedure. However, the other seven provinces in the present investigation registered statistically significant level of agreements between simulations and observations at *p* > 0.05. Furthermore, the spatiotemporal variation in LAI, AGDM, and NPP datasets were projected, revealing a typical development pattern of the other rice cultivars under environmental conditions in North Korea in the growing season (Fig. 7).

## Discussion

We applied the GOCI sensor to obtain reliable NDVI estimates for largely inaccessible rice paddy regions in North Korea. Although the GOCI-based NDVI profiles were able to efficiently reflect the growth and development of rice paddies during the summer growing season with high temporal resolution, there could be two potential limitations posed by the satellite-based crop phenology method.

Firstly, the Spectral Response Function of the red and near-infrared channel in GOCI and MODIS differ in a moderate sense because the GOCI sensor is mainly designed to monitor specifically the oceanic phenomena, and not the land surface vegetation. Consequently, the MODIS NDVI value can be somewhat larger than corresponding GOCI NDVI value. In Fig. 2, a systematic overestimation of the MODIS NDVI is observable following the decay curve after August, and this is attributable to the difference in Spectral Response Function between the GOCI and MODIS apparatus. In light of this consideration, we aver that inter-calibrations should ideally be performed to acquire a more robust relationship between the two satellites, especially for phenology-based applications considered in the present study. However, when incorporating the GOCI-based NDVI profiles into the present GRAMI model, the estimated GOCI-based NDVI profiles need to be optimised by fitting the actual crop growth and development of ground measurements. Therefore, it should be averred that the optimised GOCI-based NDVI profiles combined with a GRAMI model is potentially a likely contrivance to simulate crop yield despite slight differences in Spectral Response Function with a MODIS apparatus.

Secondly, when estimating surface reflectance from GOCI top-of-atmosphere reflectance, the ‘look-up’ table based atmospheric correction are applied to acquire the inherent spectral values of the rice paddies. The atmospherically corrected surface reflectance of GOCI are simulated using input parameters of the aerosol optical depth, water vapour, and total ozone derived from MODIS data products (i.e., MOD04, MOD05, and MOD07). However, the polar orbit MODIS sensors do not adequately cover the GOCI observation times when using the daily MODIS atmospheric product. Therefore, it is assumed that the daily variation in atmospheric constituents from MODIS meteorological product can be considerably low^32^. It is noteworthy that when a comparison of the ground station particulate matter (i.e., PM2.5) was made, the overall RMSE value of the aerosol optical depth was found to be approximately 0.123, such that the error generated in surface reflectance using MODIS daily aerosol optical depth data is expected to be less than 3% in the 6S radiative transfer model.

In respect to the reliability of the proposed approach, the areas classified as the paddy fields (measuring about 491,325 ha) in North Korea concurred with those in the reports produced by the FAO for 2016, and it was also consistent with an earlier study of Zhang *et al*.^2^. According to that FAO report, the paddy fields appear to have reduced in their relative size, from approximately 571,360 to 525,000 ha within the present study period (i.e., 2011 to 2014). In the present study, we applied stationary paddy fields due to existing data availability from COMS satellite launched in 2010. However, most satellite data with a medium-to-coarse resolution can pose some limitations in terms of determining the precise representation of the land surfaces, including reasonable identification of croplands due to limited resolution. Considering this, we are of the view that the potential differences imposed by using fixed paddy fields might be within an error range due to ground resolution issues in this study. Notwithstanding this, the removal of the pixels assigned to non-paddy fields in the simulation process could potentially minimise errors in the projected productivity. Our study also showed that more accurate classification results show the elevation, PCI, and the wetness index as the three most influential parameters.

In this study, the simulated crop growth unveiled statistically significant agreement with the observed data (refer to Fig. 5). It is noteworthy that, previously, it has been demonstrated that the incorporation of GRAMI model with remotely sensed data could support the monitoring crop productivity using operational satellites^18,33^. In this study, the GRAMI-rice version was also demonstrated to be a potential tool for regional crop monitoring purposes where it could successfully simulate the paddy productivity in North Korea (see Figs 6 and 7). In spite of the caveats in the present model, as stipulated previously, including the resilient dependence on remotely sensed data required to perform the modelling, the use of limited input parameters and variables showed its significant practical implications, particularly for unapproachable and a data-sparse region in North Korea. In such areas, the present modelling approach can be practically relevant, given that it is almost impossible to constantly monitor and simulate the crop productivity without using any operational satellite-based remote sensing data. In addition, similar practices have also been implemented using existing crop models to improve the model’s performances^17^. Huang *et al*.^17^ reported an assimilation method of satellite-based remote sensing information with a crop model used to improve the regional wheat yield estimation. Although this procedure can objectively calibrate a model to actual field conditions, it is data expensive and requires a number of model inputs, therefore, constraining the practicality of the model for real-time applications in data sparse regions.

In accordance with our findings, the dependability, portability and flexibility features of the proposed model, enshrined in its ability to utilize remote sensing approach, is a distinct advantage to solve food security issues in developing and first world nations. There is no doubt that the input requirements of our approach are less exhaustive, yet realistic, as it can utilise only the actual observations that represent environmental conditions and also, the optimisation method helps improve the simulation performance. In addition, satellite-based remote sensing is generally available for any particular region of interest on the Earth’s surface. The disadvantages, however, include the incomplete representation of remote sensing data, as well as the limited observations available during a growing season, resulting in some disagreement between simulations and observed data.

## Conclusion

Only a few previous studies have evaluated the rural environment in North Korea given that it is logistically and politically difficult to acquire scientific information on the productivity of paddy rice of the nation. Remote sensing, as advocated in this study, is considered as an efficient approach to monitor spatial regions that are largely inaccessible, and thus, applied to enhance the practical benefits of collecting such data to develop novel mechanisms that strengthen agricultural precision to addressing socio-economic issues. This study has successfully simulated the paddy productivity in North Korea using satellite-based biophysical and meteorological parameters employing the GRAMI-rice model. In summary, simulated rice yield statistically corresponded to the observed rice yield with a statistically significant range of accuracy according to t-tests (for Cheorwon at *p* = 0.183, in Paju at *p* = 0.075, and in NK at *p* = 0.101).

The practical implications of the proposed approach undertaken have far-reaching positive consequences. Our procedure in future could be used advance the science of remote sensing of the environment to solve agricultural precision issues, and supporting feasible ways to address several challenging problems facing humanity amidst, the persistent issues of climate change (e.g., drought-risk studies and capital cost reduction against measurement-based approaches in developing nations). Besides this, North Koreans today still require international relief to resolve a shortage of food despite the fact that the regime is internationally isolated. Using the results of the present study, one can not only pave a new way for regular environment monitoring and decision-support system design especially for sparse data regions where instrumental set-ups are insurmountable, but also support better agricultural policy decisions for North Korean assistance in a more effective manner.

## Materials and Method

### Study area

While North Korea exhibits a dualistic climatic influence from drylands and the ocean, the dominant climate is considerably humid continental, according to the Koppen-Geiger climate classification^3^. The summer is typically the hottest, mostly humid, and the wettest time of year. Almost 60% of all rainfall is received between June and September, indicating that the acquisition of surface spectral reflectance data of crop canopies tends to be complicated by the persistent clouds during the summer growing season.

### Satellite data

In this paper, the COMS-based data are used to retrieve biophysical and meteorological parameters of vegetation index and solar insolation from 2011 to 2014, with an aim to acquire spatiotemporal information on paddy productivity. COMS is a geostationary satellite stationed at a longitude of 128.2°E, developed by the Korea Aerospace Research Institute (KARI), launched on June 27, 2010 (https://directory.eoportal.org/web/eoportal/satellite-missions/c-missions/coms-1). It has two distinctive payloads: the GOCI and the MI sensors. GOCI is able to observe the Korean territory eight times per day with the eight solar spectral bands installed on the system. GOCI was mainly designed to monitor the ocean phenomena similar to a Sea-Viewing Wide Field-of-View Sensor (SeaWiFS) spectral waveband^34^. Meanwhile, its high temporal resolution of the observations and vegetation-sensitive spectral bands are particularly useful for land surface-based applications; and more specifically, for crop information search and monitoring^18,31^. The MI sensor is able to observe the near-real-time weather conditions using five major multispectral bands (i.e., from visible to infrared (IR) wavelengths) covering both hemispheres in Asia. In this paper, the GOCI is used to estimate the cumulative Normalized Difference Vegetation Index (NDVI) since the set-up is equipped with useful vegetation bands (i.e., Red and near infrared, NIR). On the other hand, the MI is used to estimate the solar insolation since the IR channels can be beneficial for discriminating the cloud area using characteristics of cloud low temperature. In addition, MODIS products which are polar orbit satellite are also used for two purposes. The first is the input data of atmospheric correction to estimate GOCI surface reflectance since MODIS atmospheric products showed the reasonable accuracy (https://modis-images.gsfc.nasa.gov/products.html). Secondly, the time series of MODIS-based NDVI is used as comparative analysis to show that GOCI geostationary satellite with more frequent observations can monitor the phenology of rice paddy more effectively than MODIS satellite over study area.

### GRAMI-rice Crop Model

The GRAMI-rice model, utilised in this paper, is designed to integrate remotely sensed data, allowing agricultural system modellers to simulate and monitor the potential crop growth^11^. This model can receive remote sensing data as an input, to execute the ‘within-the-season’ calibration procedure^35^, in which the simulated crop Vegetation Indices (VIs) are compared with the corresponding measured values. Four distinct parameters (i.e., leaf area index at transplanting, *L*_0_*;* the parameters in the leaf allocation function, *a* and *b*; and the parameter in the leaf senescence function, *c*) are employed in the GRAMI-rice model to describe the crop growth processes. In this study, these parameters are obtained by a Bayesian method with a prior distribution screened according to estimates from previous studies. LAI, being related to the volume of crop canopies, is three-dimensional while the reflectance of crop canopies to solar radiation is two-dimensional because the canopies of crops are the top surface of the crops. Therefore, the cubic root of LAI should be approximately proportional to the square root of reflectance. We assumed that a log-log regression model with a slope approximately 2/3 could describe the relationship between reflectance and LAI^36^. Based on this theory, the relationships between four VIs (normalized difference vegetation index, NDVI; re-normalized difference vegetation index, RDVI; optimized soil-adjusted vegetation index, OSAVI; and modified triangular vegetation index, MTVI) and the LAI were specified using the log-log linear regression models.

A Crop Information Delivery System (CIDS) was formulated by Ko *et al*.^11^, as an extended version of the GRAMI-rice model that uses images derived from remote sensing images to project the pixel-based crop growth and yield maps. CIDS employs pixel-based remote sensing data and climate data as the system’s inputs where climate data are used either a single weather station or from multiple weather stations (pixels) depending on the availability of the climate data. The GRAMI-rice model is then applied to simulate crop growth in each pixel using both types of input data. The details of these procedures and related equations are described in earlier studies by Ko *et al*.^11^. In this study, we apply the same initial conditions and parameter values used in these studies to calibrate the GRAMI-rice model.

### GRAMI-rice Model Calibration Site and Data

The present study performs a validation of the capability of the GRAMI-rice model and optimisation of the model to monitor paddy productivities using geostationary satellite imagery technique. We aim to reproduce rice productivity information in North Korea using ground truth data obtained from Cheorwon, a county region with a total area of 900 km^2^ in Gangwon province, South Korea. Cheorwon is adjacent to the border with North Korea, which experiences an annual average mean temperature of 10.2 °C and yearly average precipitation of 1,391 mm. About 74% of rainfall is concentrated between June and September, which is similar to the conditions that prevail North Korea^37^.

For the parametrisation of the GRAMI-rice model, we use a paddy yield dataset (2011 to 2014) obtained from the study site (Fig. 1b). This site is located at an experimental paddy field of the branch station of the National Institute of Crop Science (NICS). An early maturity rice cultivar, called *Odae*, bred by NICS in 1983, is transplanted in the paddy fields using thirty-day-old seedlings and applied with a correct nitrogen amount of 90 kg ha^−1^ during the growing season. Other necessary field management are practised following NICS standard cultivation method (http://www.nongsaro.go.kr). We then evaluate the GRAMI-rice model’s capability to determine the geographical crop productivity for the whole of the Cheorwon County (Fig. 1c) and the Paju city (Fig. 1d), Gyunggi province.

According to the Korean Statistical Information Service (KOSIS), the leading rice cultivar in Cheorwon is *Odae*. This cultivar is cultivated in more than 80% of the paddy fields during the study period. Rice seedlings are transplanted between May 15 and 20, which are considered as optimal growing season. All Cheorwon and Paju rice yields reported by KOSIS are used to determine the observed rice yields. The study site is carefully chosen to represent the paddy growth environments of North Korea best.

### Cumulative Crop NDVI based on the Semi-empirical BRDF Model

The GOCI red and the NIR channels are used to estimate the cumulative NDVI profiles, which are used as the crop model input parameter. Regarding the input parameters for the GRAMI-rice model, the securing of stable NDVI profiles is essential for determining the crop yield in monsoon areas. Although MODIS-based NDVI is the most widely used satellite product^38–40^, it can be challenging to obtain the continuous NDVI profiles during the summer monsoon season in the present study area^15–18,22^ since the MODIS sensor can only measure North Korea twice a day but GOCI observes 8 times a day. State-of-the-art techniques using a variety of polynomial, function-fitting, filtering, and interpolating approaches are used to interpolate and simulate missing values caused by cloud or aerosol effects. These generate reasonable estimates of vegetation phenology using optical satellites (e.g., MODIS)^41,42^. However, to more accurately reflect the actual vegetation conditions, frequent direct measurements of the crop area should be made. In this respect, we apply the GOCI sensor on the geostationary satellite instead of the polar orbit MODIS-based products.

Before estimating the NDVI profiles, atmospheric correction procedure is performed based on the tabulated values from the Second Simulation of the Satellite Signal in the Solar Spectrum (6S)^43^. The atmospherically-corrected surface reflectance of GOCI is determined using input values of the aerosol optical depth (AOD), water vapour, and the total ozone from the MODIS products (i.e., MOD04, MOD05, and MOD07). In the case of MODIS atmospheric products, the temporal resolution is daily, and the spatial resolution of AOD, water vapor, and total ozone are 10 km, 1 km, and 5 km, respectively. In order to use input data of atmosphere correction of GOCI images which have the higher spatial resolution, MODIS atmospheric products are spatially interpolated to GOCI image projection using the nearest neighbor methods. When the MODIS products are unavailable (mainly due to the cloud contaminations), we substitute the atmospheric constituents (such as AOD or water vapour based on COMS MI) from Korea Meteorological Administration (KMA) for the respective MODIS derived values^44^.

After correcting the atmospheric effects for reflectance of land surface from optical satellite, the surface bidirectional effects should be considered to obtain intrinsic reflectance properties of land surface^45^ since the surface reflectance can be changed by the relative geometry of satellite, target, and sun despite the absence of changes in rice vegetation. In other words, the surface bidirectional reflectance distribution effects are noise-like fluctuations in the time series from satellite measurements, which can cause large errors in the estimation of the phenological cycle of vegetation on a regional scale^46,47^. Therefore, it is necessary to specify the behavior of surface bidirectional effect as a function of illumination and viewing angles. To correct the surface anisotropy effects of the geostationary satellites predominantly due to the changing sun position, we use the semi-empirical bidirectional reflectance distribution function (BRDF) to estimate angular independent crop NDVI profiles with GOCI. The Ross-Thick Li-Sparse Reciprocal (RTLSR) model was used, to accord with the Roujean BRDF model that is a linear combination of three primary scattering components: isotropic scattering, volumetric scattering, and geometric scattering^47,48^:

1 $${\rm{\rho }}({\theta }_{s},{\theta }_{v},\varnothing )={f}_{iso}+{f}_{geo}{k}_{geo}({\theta }_{s},{\theta }_{v},\varnothing )+{f}_{vol}{k}_{vol}({\theta }_{s},{\theta }_{v},\varnothing )$$

where *f*_*iso*_, *f*_*geo*_, and *f*_*vol*_ are the spectrally dependent model parameters, *f*_*iso*_ is the Lambertian reflectance in the nadir direction, *f*_*geo*_ is the coefficient of the LiSpare-Reciprocal geometric kernel k_*geo*_, and *f*_*vol*_ is the coefficient of the Ross-Thick volumetric kernel k_*vol*_. It is important to note that the k_*geo*_ and k_*vol*_ are kernel functions of the viewing zenith angle *θ*_*v*_, the solar zenith angle *θ*_*s*_, and the relative azimuth angle $\varnothing$, and these provide shapes for volumetric and geometric-optical scattering BRDFs.

The BRDF model kernel coefficients are estimated independently for each gridded pixel location by the inversion of Eq. (1) using GOCI surface reflectance values with sensor-target-solar geometry sensed during the 16-day composite period^49–51^. In this study, BRDF Adjusted Reflectance (BAR) for GOCI is also estimated from Eq. (1) but using its fixed viewing angle and mean solar zenith angle without adjusting the viewing zenith angle to the nadir direction. This is done because the GOCI sensor is not able to acquire the nadir direction angular sampling over the study area^52^.

In the case of MODIS Nadir BRDF-Adjusted Reflectance (MCD43) products from NASA, it is estimated from Eq. (1) to normalise the nadir view angles and the mean solar zenith angle using MODIS surface reflectance products (MOD09A1 and MYD09A1). MODIS operational NBAR products (MCD43) from NASA were retrieved every 8 days because of the archival constraints^50,51^. In this study, the BRDF adjusted reflectance (BAR) values from GOCI are also estimated by a daily rolling strategy over a 16-day retrieval period to obtain a more intuitive interpretation of phenology characteristics and to capture more subtle details^53,54^.

### Insolation from COMS MI based on the physical model

In this study, we estimate the incident solar radiance on the surface (insolation), which is a primary meteorological parameter, using the COMS MI to reflect the energy source of photosynthesis on crop canopies. Of various satellite-based insolation models^55–57^, the pixel-based physical model is adjusted using instantaneous satellite observations and atmospheric information. The adjustment is made because it was difficult to interpret the radiation effects of atmospheric constituents and cloud under the hemispherical sphere condition due to complex physical characteristics and time-consuming calculations^58,59^.

The calculation of the satellite-based solar insolation with COMS MI is based on the Kawamura physical model^58^, but the model had an improved cloud factor as it considered the satellite visible reflectance and the solar zenith angle instead of the brightness temperature because the pass depth of cloud is more sensitive to the amount of irradiance attenuation^60^. The details of the physical model are as follows^58,60–62^:

2 $${S}_{T}={S}_{I}+{S}_{R}+{S}_{A}$$

where *S*_*T*_, *S*_*I*_, *S*_*R*_ and *S*_*A*_ are the total solar insolation, direct irradiance, diffuse irradiance due to Rayleigh scattering, and the diffuse irradiance due to scattering by aerosols, respectively.

After determined atmospheric parameterisation as above equation, the cloud discrimination is performed using visible and IR channels since the process of the physical model is different whether the cloud exists or not. In the case of unclouded sky condition, the determined atmospheric parameterisation is used as clear sky solar insolation. Under cloudy sky condition, while, Eq. (3) is used with cloud factor for total insolation to reflect the radiation attenuation by cloud.

3 $${S}_{T}=({S}_{I}+{S}_{R}+{S}_{A})\times Cloud\,Factor$$

where *Cloud Factor* is the ratio of attenuation by cloud for incident solar radiation using COMS MI visible band and solar zenith angle^61^. The cloud top reflectance from COMS MI is observed to indirectly determine the cloud optical thickness, meaning that the higher cloud top reflectance shows more incident radiation which is scattered into the atmosphere by clouds. Also, the solar zenith angle over the dark area is used to determine cloud effects, because the attenuation of incident solar radiation by clouds depends on the pass depth of the clouds^61^. Detailed definitions of those parameters used for the physical model of the satellite-based solar insolation are available in previous studies^60,61^.

### Air temperature from KLAPS based on the numerical model

The air temperatures from the Korea local analysis and prediction system (KLAPS) are utilised to simulate the rice production as temperature is a driving variable to determine plant phenology for the crop model. The KLAPS is designed to forecast weather conditions of the Korean peninsula with a pixel resolution of 5 km and forecast frequency of 24 times every hour for the period a day^63^. The KLAPS produces high-resolution (1.5 km) reanalysis data based on its analysis scheme using all possible measured weather data from the region of interest^63^. The KLAPS also adapts the data assimilation part of the local analysis and prediction system (LAPS) developed by the US National Oceanic and Atmospheric Administration/Forecast Systems Laboratory (NOAA/FSL) and is classified into both data collection and analysis modules. The analysis process comprises of the surface analysis procedures, as well as 3-dimensional wind, temperature, humidity, cloud, precipitation, and soil analysis procedures. Further details of the KLAPS procedure are found in Albers^64^, Albers *et al*.^65^, and McGinley *et al*.^66^.

### Crop classification and acreage map of North Korea

Paddy fields are classified using several topographical variables and the GOCI NDVI phenological parameters as described (Fig. 1b). Using median values of the 4-year daily GOCI NDVI data (2011 to 2014), the paddy classification index (PCI) are calculated as follows:

4 $${\rm{PCI}}=\frac{(NDV{I}_{harvest}-NDV{I}_{transplant})}{(NDV{I}_{harvest}+NDV{I}_{transplant})}$$

where NDVI_harvest_ and NDVI_transplant_ represent the NDVI values at harvest (~day of year 270) and the NDVI values at transplanting (~day of year 140). Topographical variables used were elevation, slope degree, and wetness index. These were derived from 30-m ASTER digital elevation model provided by the U.S. Geological Survey (http://earthexplorer.usgs.gov). The variables were calculated using the terrain analysis modules as per the SAGA GIS program^67^.

The Random Forest (RF) algorithm is used to classify the paddy field areas, which is an ensemble learning algorithm combining a set of classification trees^68^. RF is an effective method for remote sensing applications due to the ease of model construction and the reliable accuracy of its classification^69^. The size of the variable subset of RF is tuned using the R package “caret”^70^. The number of decision trees is kept reasonably high (~1,000) for optimal feature identification. For variable selection purposes, the recursive feature elimination (RFE), which measures the variable importance and then repetitively eliminates the least important variables one at a time until the best variable is left, is used^71^.

In this task, the package “caret” provided the functions to implement RFE procedure^70^. A resampling method for the RFE procedure is practised with five repetitions and ten-fold cross-validation. The variables selected via RFE are found to be, in order of wetness index, elevation, paddy classification index, maximum peak value, maximum fading date, maximum peak date, maximum fading rate, slope degree, and maximum growth rate. For calibration, a reference dataset for paddy field classification is constructed using the land use map of northern provinces (Kangwon and Gyeonggi) in South Korea (http://egis.me.go.kr). As North Korea is inaccessible for ground truth data, this study uses an available digital map of paddy field areas in South Hwanghae for validation, which is classified with an on-screen digitisation method using high spatial resolution KOMPSAT-3, 3A, and RapidEye images.
